# An extended Hegselmann-Krause model incorporating agent heterogeneity and influence propagation

**DOI:** 10.1371/journal.pone.0334059

**Published:** 2025-10-28

**Authors:** Fei Liu, Zhili Liu, Hua Zhou

**Affiliations:** 1 School of Computer Science and Engineering, Hunan University of Information Technology, Changsha, Hunan, China; 2 School of Information and Mechatronic Engineering, Hunan International Economics University, Changsha, Hunan, China; 3 School of Computer Science and Engineering, Hunan University of Information Technology, Changsha, Hunan, China; AGH University of Krakow: Akademia Gorniczo-Hutnicza im Stanislawa Staszica w Krakowie, POLAND

## Abstract

Traditional models of opinion dynamics provide a simplified framework for understanding human behavior in basic social scenarios. However, with the rise of complex communication patterns and heterogeneous social interactions in modern networks, more comprehensive and nuanced models are required. This paper proposes an extended opinion dynamics model that integrates individual heterogeneity, homophily-based influence weights, and multi-layer influence propagation mechanisms. First, we modify the classical Hegselmann-Krause (HK) model by introducing a selective influence neighborhood based on individuals’ social network connections, thereby capturing the structure-dependent nature of interpersonal interactions. Second, drawing on the theory of homophily, we model the influence weights between individuals according to their opinion similarity and domain-specific attributes. Third, we incorporate a k-layer influence propagation mechanism to simulate indirect social influence through extended paths in the network. Finally, simulation experiments and validation using real-world data demonstrate that the proposed model effectively captures the dynamics of opinion evolution and enhances predictive accuracy in complex social systems.

## 1 Introduction

Opinions, as individuals’ perceptions and judgments of various matters, are pervasive in social interactions, public discourse, and decision-making processes [1]. These opinions are shaped not only by personal experience and cognitive structures but also evolve dynamically through social interactions and media dissemination, exhibiting complex behavioral patterns [2,3]. To investigate the mechanisms underlying opinion formation and evolution, researchers have proposed a range of opinion dynamics models—such as the Hegselmann-Krause (HK) model [4], the Deffuant-Weisbuch (DW) model [5], and the DeGroot model [6]—which incorporate factors including individual attributes, social influence, and network topology. Building on these foundations, subsequent studies have examined the roles of different types of individuals, such as opinion leaders [7], stubborn agents [8], and heterogeneous participants [9], highlighting their critical influence in the opinion dissemination process. Moreover, empirical and theoretical analyses have demonstrated that network connectivity [10] and structural characteristics [11] significantly affect the speed of opinion diffusion and the emergence of consensus.

With the rapid development of the Internet and social media, the modes and speed of information dissemination have undergone profound changes. Interactions among individuals have become increasingly frequent, resulting in unprecedented complexity in the evolution of public opinion [12,13]. For example, on social networking platforms, users can express their views through actions such as liking, commenting, and sharing, and are simultaneously influenced by others’ opinions during these interactions, forming a dynamic and nonlinear opinion evolution process [14,15]. At the same time, the wider reach and accelerated speed of information dissemination have led to high uncertainty and diversity in the diffusion and transformation of opinions [16–19]. Moreover, the spread of misinformation and extreme viewpoints has further complicated the public opinion landscape, posing new challenges to social governance and public decision-making [20]. Consequently, traditional opinion dynamics models often fall short in capturing the complex and evolving mechanisms of opinion formation and propagation in today’s diversified and dynamic social environments.

To address the aforementioned challenges, researchers have extended and refined classical opinion dynamics models from multiple perspectives [21–23]. On one hand, scholars have enhanced the classification of agents within the models to explore the roles of different types of individuals in the opinion dissemination process. For instance, Zhang [24] investigated how the emotions of opinion leaders influence the stock prices of peer companies, and further analyzed the patterns and mechanisms of emotional transmission in online forums. Xin [25], from a systems and control perspective, examined the dynamic behaviors of agents with stubborn characteristics in social networks. In addition, some studies have categorized individuals based on emotional types [26] or opinion types [27], enabling more fine-grained analysis. On the other hand, research has increasingly focused on the structural characteristics of networks among individuals. Features such as the small-world effect and scale-free property of complex networks have been shown to significantly facilitate rapid information dissemination, especially when the network includes a few highly connected “hub” nodes capable of broadcasting information across the entire system [28]. Moreover, community structures also play a critical role in shaping intra-group opinion consensus. Studies have found that different communities often form localized agreements, which may exacerbate inter-group polarization and hinder the formation of global consensus [29]. Meanwhile, factors such as individual interaction patterns [30], group heterogeneity [31], and modes of information transmission [32] also exert substantial influence on the process of opinion evolution. These findings reveal the diverse mechanisms through which network structures regulate group opinion dynamics, laying a solid theoretical foundation for understanding information dissemination, public opinion formation, and collective decision-making in networked environments.

In the current context, research on opinion dynamics reveals two prominent and closely interconnected trends. First, with the continuous diversification and enrichment of information dissemination channels, individuals’ modes of expressing opinions have expanded beyond traditional face-to-face communication to encompass various platforms such as social media, online forums, and instant messaging. This multiplicity of expression channels significantly complicates the opinion evolution process, necessitating careful consideration of domain-specific characteristics and homogeneity effects within groups. Second, interpersonal influence is no longer confined to direct neighbors or acquaintances but propagates through complex multi-path structures within social networks, where influence can be amplified or attenuated at successive stages. This non-local influence mechanism endows opinion dynamics with multi-scale and multi-level temporal features, greatly increasing the complexity and realism of modeling efforts. Motivated by these trends, this paper proposes a comprehensive opinion dynamics model integrating individual heterogeneity, domain attributes, and multi-path influence propagation mechanisms, aiming to establish a more systematic and precise theoretical framework. This model seeks to uncover the intrinsic patterns and dynamic mechanisms of opinion evolution in the information era, thereby providing effective analytical tools and theoretical support for understanding the formation of complex public opinions in modern society.

The remainder of the paper is organized as follows: In Sect 2, some fundamental definitions and preliminaries are briefly reviewed. In Sect 3, the opinion evolution model is proposed by considering both individual heterogeneity and influence propagation. In Sect 4, simulation experiments and sensitivity analyses are conducted to verify the effectiveness and validity of the proposed model. Finally, in Sect 5, some significant conclusions are drawn, and potential directions for future research are discussed.

## 2 Preliminaries

In this section, some related definitions are briefly reviewed, which are necessary to the subsequent analysis.

### 2.1 Social network

The social network is modeled as a time-varying graph, capturing the structural relationships among agents. The components of the network are defined as follows:

**(1) Network structure:** At time t∈ℕ, the social network is represented by a directed graph ${𝒢}^{t}=(𝒱,{ℰ}^{t})$, where: $𝒱=\{v_{1},v_{2},\ldots ,v_{N}\}$ denotes the set of *N* agents (nodes); ${ℰ}^{t}\subseteq 𝒱\times 𝒱$ denotes the set of edges at time *t*, representing the connections among agents.

**(2) Link matrix:** Let $E=(e_{ij}^{t}{)}_{N\times N}$ denote the link matrix of the network at time *t*, where $e_{ii}^{t}=0$ for all i=1,…,N, ensuring no self-links between agents. For i,j=1,…,N and i≠j , the entries of $e_{ij}^{t}$ are defined as:


$$e_{ij}^{t}=\left\{ \begin{array}{cc} 1, & \text{if there exists a link from }v_{i}\text{ to }v_{j},\\ 0, & \text{otherwise.} \end{array}\right.$$

**(3) Neighborhood:** For each agent $v_{i}\in 𝒱$, the influence neighborhood at time *t* is defined as ${𝒬}_{i}^{t}=\left\{v_{j}\in 𝒱\;|\;e_{ij}^{t}=1\right\}$, the set of agents to whom $v_{i}$ has outgoing links at time *t*. Thus, the collection of all individual neighborhoods can be denoted by ${𝒬}^{t}=\left\{{𝒬}_{1}^{t},{𝒬}_{2}^{t},\ldots ,{𝒬}_{N}^{t}\right\}$.

### 2.2 Barabási-Albert model

The Barabási–Albert (BA) model generates scale-free networks characterized by a power-law degree distribution, capturing key structural features of real-world complex systems. The network construction process is defined as follows:

**(1) Initial Condition:** Begin with a fully connected network of *m*_0_ nodes, denoted by ${𝒢}^{0}=({𝒱}^{0},{ℰ}^{0})$, where $|{𝒱}^{0}|=m_{0}$, and each node $v_{i}\in {𝒱}^{0}$ satisfies $\deg(v_{i})=m_{0}-1$.

**(2) Network Growth:** At each discrete time step t=1,2,…, a new node $v_{m_{0}+t}$ is added to the network. This new node forms *m* links to existing nodes, where $1\leq m\leq m_{0}$.

**(3) Preferential Attachment:** The probability *P*(*i*) that the new node connects to an existing node $v_{i}\in {𝒱}^{t-1}$ is proportional to the degree $k_{i}^{t-1}=\deg(v_{i})$ at time *t*–1, and is given by:


$$P(i)=\frac{k_{i}^{t-1}}{\sum_{v_{j}\in {𝒱}^{t-1}}k_{j}^{t-1}}.$$

**(4) Iteration:** Repeat steps 2 and 3 until the network reaches the target size of *N* nodes, i.e., until $|{𝒱}^{t}|=N$, where *N*>*m*_0_.

### 2.3 Classic HK model

The Hegselmann–Krause (HK) model is a foundational framework in opinion dynamics, describing how agents iteratively adjust their opinions through local interactions constrained by bounded confidence.

Let $𝒱=\{v_{1},v_{2},\ldots ,v_{N}\}$ denote the set of agents. Each agent $v_{i}\in 𝒱$ holds a continuous opinion $x_{i}^{t}\in [0,1]$ at discrete time t∈ℕ. The bounded confidence parameter is denoted by ε∈[0,1], representing the maximum opinion difference tolerated for interaction. At time step *t* + 1, the opinion of agent $v_{i}$ is updated as:

$$x_{i}^{t+1}=\sum_{v_{j}\in ℐ(i,x_{i}^{t})}\alpha_{ij}^{t}\cdot x_{j}^{t}$$ (1)

where: $ℐ(i,x_{i}^{t})=\left\{v_{j}\in 𝒱\;|\;|x_{i}^{t}-x_{j}^{t}|\leq \varepsilon \right\}$ is the set of agents whose opinions lie within the confidence interval of $v_{i}$, $\alpha_{ij}^{t}\in [0,1]$ denotes the influence weight of agent $v_{j}$ on agent $v_{i}$, subject to the normalization constraint $\sum_{j=1}^{N}\alpha_{ij}^{t}=1$.

To better reflect selective influence in structured social networks, an additional constraint is imposed: agent $v_{j}$ must also belong to the connection neighborhood ${𝒬}_{i}^{t}\subseteq 𝒱$ of agent $v_{i}$, as defined in the social network model. Under this modification, the opinion update rule becomes:

$$x_{i}^{t+1}=\sum_{v_{j}\in ℐ(i,x_{i}^{t})\cap {𝒬}_{i}^{t}}\alpha_{ij}^{t}\cdot x_{j}^{t}$$ (2)

This extension captures the fact that social influence is often constrained by relational boundaries, and agents are more likely to be affected by peers within their interaction neighborhood or social circle.

## 3 A novel HK model incorporating agent heterogeneity and influence propagation

### 3.1 Modeling influence weights based on agent heterogeneity

According to homophily theory, individuals are more likely to form connections with others who share similar characteristics. Based on this principle, we propose that the influence exerted by agent $v_{j}$ on agent $v_{i}$ depends jointly on their opinion similarity and the similarity of their personal attributes. Formally, the influence score $S_{ij}^{t}$ is defined as follows:

**(1)** If $v_{j}\in ℐ(i,x_{i}^{t})\cap {𝒬}_{i}^{t}$, then

$$S_{ij}^{t}=clip\left(\omega_{1}\cdot \left(1-\left\|x_{i}^{t}-x_{j}^{t}\right\|\right)+\omega_{2}\cdot \left(1-\left\|C_{i}^{t}-C_{j}^{t}\right\|\right),\;0,\;1\right)$$ (3)

**(2)** If $v_{j}\notin ℐ(i,x_{i}^{t})\cap {𝒬}_{i}^{t}$, then

$$S_{ij}^{t}=0$$ (4)

Here, $S_{ij}^{t}\in [0,1]$ represents the influence score of agent $v_{j}$ on agent $v_{i}$, while $\omega_{1},\omega_{2}\in [0,1]$ are the weights assigned to opinion similarity and attribute similarity, subject to $\omega_{1}\;+\;\omega_{2}=1$. The notation ‖·‖ measures the difference between agents: the opinion difference is defined as $\left\|x_{i}^{t}-x_{j}^{t}\|=|x_{i}^{t}-x_{j}^{t}\right|$, and the attribute difference is given by:

$$\|C_{i}^{t}-C_{j}^{t}\|=\frac{\sum_{d=1}^{\left|C\right|}|c_{di}^{t}-c_{dj}^{t}|}{\left|C\right|}$$ (5)

where |C| denotes the number of dimensions in the attribute vector $C_{i}^{t}$, and $c_{di}^{t}$ is the value of the *d*-th feature of agent $v_{i}$ at time *t*. The function clip(·,0,1) ensures that the influence score remains within the interval [0,1], avoiding extreme values.

It is worth noting that $\omega_{1}$and $\omega_{2}$ represent the relative importance of opinion similarity and attribute similarity. In particular, when $\omega_{1}=1,$ the influence score depends solely on opinion similarity, whereas when $\omega_{2}=1$, it is determined entirely by attribute similarity. This formulation not only ensures a normalized balance between the two factors but also provides a flexible mechanism for analyzing how different weight settings affect the overall influence dynamics in subsequent sections.

Subsequently, the normalized influence weight $\alpha_{ij}^{t}\in [0,1]$ is computed as:

$$\alpha_{ij}^{t}=\frac{S_{ij}^{t}}{\sum_{\begin{array}{c} k=1\\ k\neq i \end{array}}^{N}S_{ik}^{t}},\;i,j=1,2,\ldots ,N$$ (6)

This formulation captures both homophily in social networks and the bounded rationality of opinion updates, ensuring that agents are selectively influenced by both like-minded and socially proximate peers.

**Fig 1:**
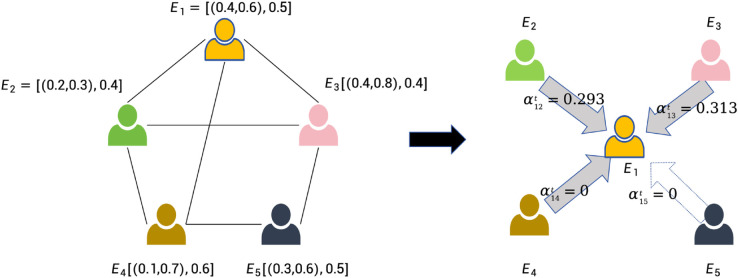
Illustration of homophily-based influence at time t: Arrows indicate the presence of connections, and $E_{i}=[(c_{d1}^{t},c_{d2}^{t}),x_{i}^{t}],i=1,2,\ldots ,5$ denotes the feature and opinion values of the five agents at time *t.*

**Example 1**: Consider the social network in Fig 1.

At time *t*, agent $v_{1}$ is influenced by other agents, assuming the opinion threshold ε=0.2. To illustrate the computation of $S_{12}^{t}$, suppose each agent is characterized by two personal attributes. Let the attribute vectors be *C*_1_ = [0.4,0.6] and *C*_2_ = [0.2,0.3], and let the opinion values be $x_{1}^{t}=0.5$, $x_{2}^{t}=0.4$. Assume the weight vector is W=(0.5,0.5). Then, the influence score of agent $v_{2}$ on agent $v_{1}$ at time *t* is given by:


$$S_{12}^{t}=\text{clip}\left(0.5\times \left(1-|0.5-0.4|\right)+0.5\times \left(1-\frac{\left|0.4-0.2|+|0.6-0.3\right|}{2}\right),\;0,\;1\right)\;=\text{clip}\left(0.5\times 0.9+0.5\times (1-0.25),\;0,\;1\right)\;=\text{clip}\left(0.45+0.375,\;0,\;1\right)\;=\text{clip}(0.825,\;0,\;1)=0.825$$

Similarly, $S_{13}^{t}=0.85,S_{14}^{t}=0.9$. Since $v_{5}\notin Q_{1}^{t}$, $S_{15}^{t}=0$. After that, the weight that other agents influences $v_{1}$ can be calculated as:


$$\alpha_{12}^{t}=0.825/(0.825+0.85+0.9)=0.32,\alpha_{13}^{t}=0.33,\alpha_{14}^{t}=0.35$$

### 3.2 Influence transmission mechanism in networked interactions

**Fig 2:**
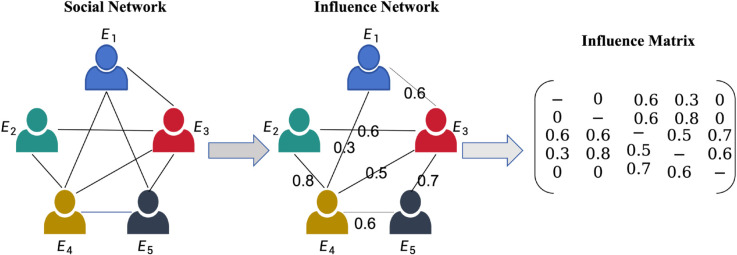
Influence network at time *t*: Arrows indicate directed influence, and numbers on the arrows represent the strength of influence at time *t.*

Social network analysis explores the relationships among social entities and has found broad applications across numerous disciplines. Building upon this, researchers have extended the analysis to focus on influence networks, which capture how individuals affect each other’s judgments or decisions. At a given time step, the influence exerted by one agent on another can be quantified and represented through an influence matrix, as illustrated in Fig 2.

A growing trend in information aggregation highlights that individuals increasingly rely on mutual influence to determine the weight of others’ opinions before integrating them. Wu [33] emphasizes that interpersonal influence should be considered during agent interactions and proposes the concept of dual influence propagation: the influence between two individuals can be shaped indirectly through their mutual connections. To more effectively synthesize the opinions of experts, we extend this idea by incorporating multi-step influence propagation, allowing indirect influence paths to be systematically integrated into the aggregation process.

At time *t*, suppose the direct influence among agents is represented by a matrix *G*, defined as:


$$G=\left[\begin{array}{cccc} - & \alpha_{12}^{t} & \cdots & \alpha_{1N}^{t}\\ \alpha_{21}^{t} & - & \cdots & \alpha_{2N}^{t}\\ \vdots & \vdots & \ddots & \vdots \\ \alpha_{N1}^{t} & \alpha_{N2}^{t} & \cdots & \alpha_{NN}^{t} \end{array}\right]$$

where $\alpha_{ij}^{t}$ (for i,j=1,2,…,N, i≠j) represents the degree to which agent $v_{i}$ is directly influenced by agent $v_{j}$ at time *t*. If there is no influence relationship between $v_{i}$ and $v_{j}$, then $\alpha_{ij}^{t}=0$.

To mitigate the disparity in evaluation intensity among agents, the direct influence matrix *G* is normalized to obtain a stochastic matrix $R=[r_{ij}{]}_{N\times N}$, defined by:

$$R=\frac{1}{\max_{1\leq i\leq N}\sum_{j=1}^{N}\alpha_{ij}^{t}}\cdot G$$ (7)

After normalization, $0\leq r_{ij}\leq 1$, and the matrix powers satisfy $\lim_{k\to \infty }R^{k}=0$.

When computing the indirect influence between $v_{i}$ and $v_{j}$, two scenarios are considered:

(i) If $v_{i}$ and $v_{j}$ are connected via a common intermediary $v_{k}$, their indirect influence is measured by $r_{ik}\cdot r_{kj}$.
(ii) Even if there is no direct path via $v_{k}$ (i.e., *r*_*ik*_ = 0 or *r*_*kj*_ = 0), the product $r_{ik}\cdot r_{kj}=0$ still represents the potential indirect influence via $v_{k}$.

Based on this, the dual-layer indirect influence between $v_{i}$ and $v_{j}$ is given by $\sum_{k=1}^{N}r_{ik}\;\cdot \;r_{kj}$, which corresponds to the square of the influence matrix: $R^{2}=R\;\cdot \;R$. As a result, triple-layer influence is expressed as $R^{3}=R\cdot R^{2}$, and in general, the *k*-layer influence matrix is *R*^*k*^.

The comprehensive influence matrix $T=[t_{ij}{]}_{N\times N}$ aggregating all indirect influence across infinite layers can then be computed as:

$$T=R+R^{2}+R^{3}+...=\sum_{k=1}^{\infty }R^{k}=R(I-R{)}^{-1}$$ (8)

**Remark 1:** Since this section focuses on the analysis at a specific time step, we define the direct influence matrix *G* and the stochastic matrix $R=[r_{ij}{]}_{N\times N}$ without explicitly attaching time indices, for notational simplicity. It should be noted, however, that both matrices are inherently time-dependent in the dynamic process.

### 3.3 Construction of the opinion evolution model

**Fig 3:**
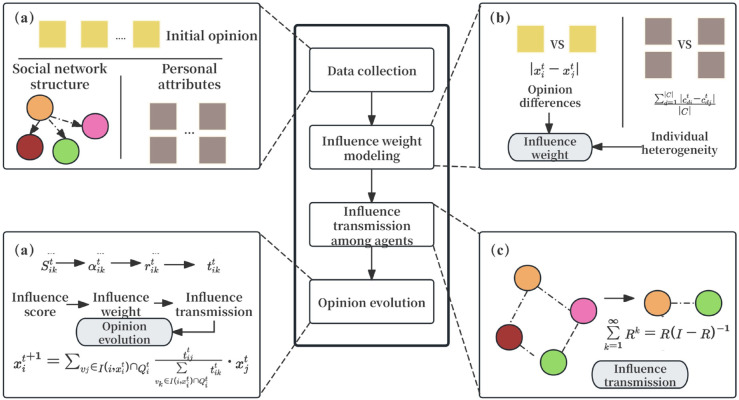
Framework of the proposed opinion evolution model: a,b,c and d represent the four modules of the model.

Building upon the extended HK framework and the influence propagation mechanism, we now formulate a comprehensive opinion evolution model that integrates both agent heterogeneity and multi-layer influence dynamics. This model reflects how individuals update their opinions not only based on direct interactions constrained by bounded confidence and social connectivity, but also by incorporating indirect influence pathways embedded in the network structure. The updated opinion dynamics equation is defined as follows:

$$x_{i}^{t+1}=\sum_{v_{j}\in I(i,x_{i}^{t})\cap Q_{i}^{t}}\frac{t_{ij}^{t}}{\sum_{v_{k}\in I(i,x_{i}^{t})\cap Q_{i}^{t}}t_{ik}^{t}}\cdot x_{j}^{t}$$ (9)

where $I(i,x_{i}^{t})\;\cap \;Q_{i}^{t}$ defines the set of neighbors influencing individual *i* at time *t*, requiring not only opinion similarity but also belonging to a specific social circle, $t_{ij}^{t}$ incorporates both individual heterogeneity and the transmissibility of influence.The overall flow of this modeling framework is illustrated in Fig 3

To quantitatively assess the dynamics and outcomes of the proposed opinion evolution model, we introduce two key metrics: Convergence Speed and Clustering Number. These indicators reflect the efficiency of opinion stabilization and the degree of polarization or consensus formation in the population, respectively.

**(1) Convergence Speed (*T*_*c*_):** This metric measures the time required for the system to reach a stable state, defined as the minimum time step *t* such that for all individuals *i*, their opinions satisfy $|x_{i}^{t+k}\;-\;x_{i}^{t}|\leq \delta$ for any k≥K. The parameter *K* ensures stability over consecutive time steps, and the inclusion of *δ* effectively overcomes the influence of oscillating nodes.

$$T_{c}=\min\left\{t|\forall i,\;|x_{i}^{t+k}-x_{i}^{t}|\leq \delta ,\;\forall k\geq K\right\}$$ (10)

**(2) Clustering Number (*N*_*c*_):** This metric quantifies the number of distinct opinion clusters formed upon convergence. Each cluster *C*_*k*_ consists of individuals *i* who satisfy $|x_{i}-x_{j}|\leq \varepsilon$ for any two individuals *i* and *j* within the cluster, where *ε* is the clustering threshold.

$$N_{c}=\left|\left\{C_{k}|C_{k}=\left\{i|\forall j\in C_{k},\;|x_{i}-x_{j}|\leq \varepsilon \right\}\right\}\right|$$ (11)

It is worth noting that *ε* also functions as the interaction threshold, whereby individuals within this range are regarded as belonging to the same cluster. Consequently, the same parameter value is deliberately employed for both the bounded confidence and clustering definitions.

These two metrics provide an interpretable means to assess both the efficiency and the diversity of opinion formation under various influence mechanisms and model parameters.

## 4 Numerical simulation

This section conducts numerical simulations to evaluate the effectiveness and realism of the proposed opinion dynamics model. In addition, the simulations explore how changes in the initial opinion mean, initial opinion variance, weight allocation, and group size affect the speed of opinion convergence and the resulting number of opinion clusters.

### 4.1 Simulation algorithm and parameter settings

Since BA model effectively captures the scale-free nature of real-world social networks, it is employed to simulate the structural topology of agent interactions. Therefore, the overall algorithm is shown in Algorithm 1. Detailed parameter settings are shown in Table 1, unless otherwise specified.

**Table 1:** Experimental parameter settings.

Parameter	Description	Value or Distribution
*N*	Group size	200
*ε*	Confidence bound level	0.1
*μ*	Mean of initial opinions	0.5
$\sigma^{2}$	Variance of initial opinions	0.2
$x_{i}^{0}$	Initial individual opinions	$x_{i}^{0}\sim 𝒩(\mu ,\sigma^{2})$
$c_{i}^{0}$	Personal attribute values	$c_{i}^{0}\sim 𝒩(0.5,0.1)$
*K*	Minimum convergence step number	*K* = 5
*δ*	Convergence threshold	δ=0.1
$T_{\text{max}}$	Maximum number of iterations	200

**Algorithm 1. Hegselmann-Krause model with agent heterogeneity and influence propagation.**



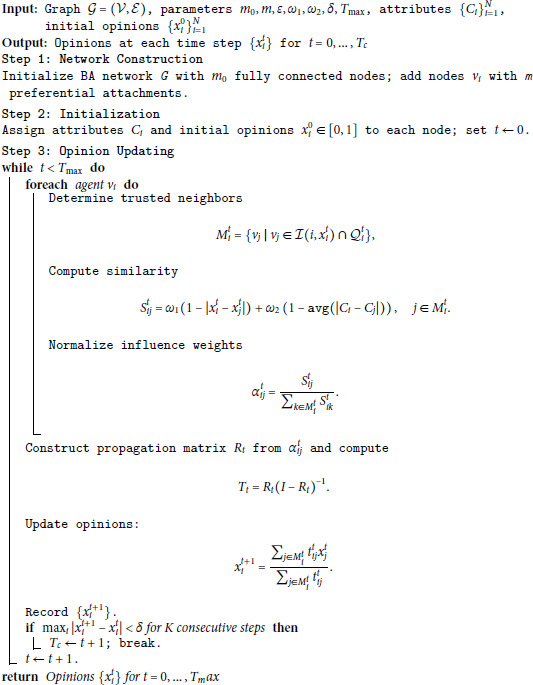



### 4.2 Comparative simulation analysis

**Fig 4:**
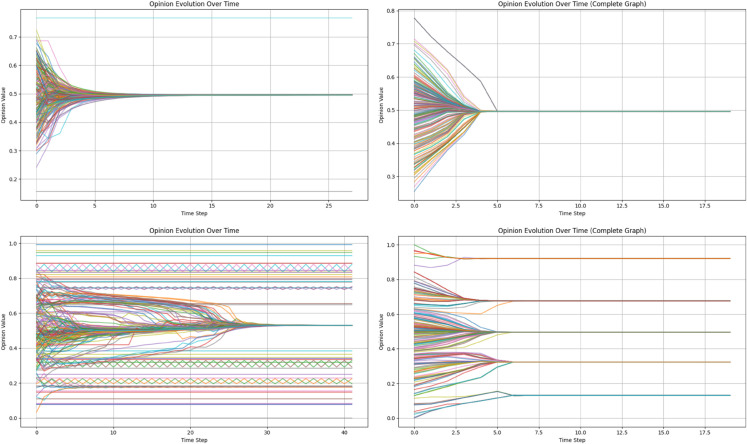
Comparison of opinion dynamics in traditional and proposed models: Top left: opinion dynamics of HK model in the BA network; Top right: opinion dynamics of HK model in the connected network; Bottom left: opinion dynamics of the proposed model in the BA network; Bottom right: opinion dynamics of the proposed model in fully connected network.

To verify the effectiveness of the proposed model and to justify the choice of the BA network topology, we performed comparative simulations under identical initial conditions, as shown in Fig 4.

As illustrated in Fig 4, several observations can be made: (1) Regardless of the network structure, the traditional HK model reaches consensus relatively quickly and typically produces a small number of opinion clusters compared with the proposed model. (2) When comparing the BA network with the fully connected network, opinion evolution in both models appears simpler in the fully connected topology. (3) The proposed model leads to the emergence of multiple opinion clusters rather than a single unified consensus, along with persistent opinion oscillations in some agents.

These results can be explained by both the design of the proposed model and the structural characteristics of the BA network. First, the proposed model incorporates agent heterogeneity and an influence propagation mechanism, which together capture more realistic social interaction patterns. Heterogeneity in individual attributes leads to differentiated trust and influence weights, reducing the uniformity of interactions and slowing down consensus formation. The multi-step influence propagation allows opinions to spread indirectly through neighbor chains, enabling minority viewpoints to persist and increasing the likelihood of multiple clusters. Dynamic and adaptive influence weights further contribute to the emergence of oscillatory behaviors in some agents.

Second, the choice of the BA network is motivated by its scale-free property and heterogeneous degree distribution, which resemble many real-world social networks. High-degree hubs play a disproportionate role in opinion transmission, accelerating convergence in certain clusters while preserving diversity in others. In contrast, the fully connected network represents a homogeneous interaction structure, leading to simpler and more uniform opinion dynamics. Together, these findings validate the effectiveness of the proposed model in capturing complex opinion evolution and demonstrate the suitability of the BA topology for simulating realistic social influence processes.

Beyond theoretical explanations, these dynamics also resonate with real-world opinion formation patterns. For instance, in today’s increasingly complex and digitally mediated social environments, public discourse on issues such as data privacy, algorithmic fairness, or public health interventions often leads to persistent opinion fragmentation. Individuals are embedded in overlapping online and offline networks, where interactions are shaped not only by opinion proximity but also by shared professional, cultural, or ideological affiliations. The proposed model, by incorporating both social connectivity and attribute-based similarity, effectively captures this layered interaction structure. As such, it offers a more realistic account of how diverse and polarized opinion clusters emerge and persist in modern, interconnected societies.

### 4.3 Scalability analysis

To further investigate the robustness and generalizability of the proposed opinion dynamics model, this section explores how the system responds to changes in key structural and cognitive parameters. Specifically, we examine the influence of the initial opinion mean and variance on the evolution and final distribution of opinions. Moreover, we analyze how simultaneous variations in these two factors affect convergence behavior and opinion clustering. Finally, we assess the impact of group size on the model’s scalability, focusing on how larger populations shape the speed of convergence and the resulting diversity of opinion clusters.

**(1) Impact of initial opinion mean on the evolution**

**Fig 5:**
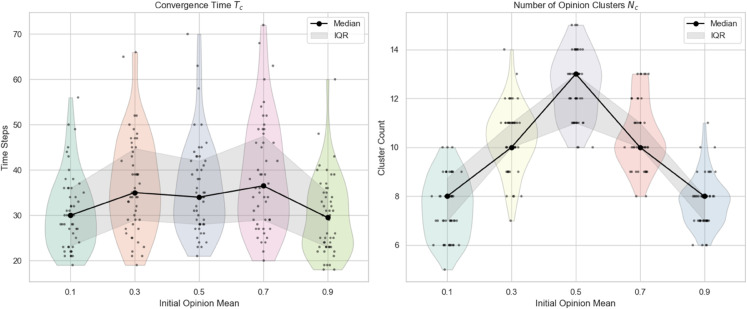
Comparison of convergence speed and cluster count under different initial opinion means: The black dots represent the median of 100 repeated trials, and the shaded area represents the interquartile range (IQR).

This part investigates the impact of the initial opinion mean on the opinion evolution process while keeping other model parameters fixed. By setting the initial mean values to 0.1, 0.3, 0.5, 0.7, and 0.9, we conducted 100 independent simulation runs for each scenario. The convergence speed and the resulting number of opinion clusters were recorded and analyzed. Fig 5 illustrates how varying the initial opinion mean influences the dynamics of the model, highlighting the critical role of the initial opinion distribution center in shaping collective opinion formation.

From Fig 5, it can be observed that the overall convergence speed of the group remains relatively stable and does not significantly vary with changes in the initial opinion mean. In contrast, the number of opinion clusters exhibits a non-monotonic trend: it initially increases as the initial opinion mean rises, reaches a peak, and then decreases.

This phenomenon can be explained by the interplay between opinion distribution and influence dynamics. When the initial opinion mean is low or high (close to the boundaries of the opinion spectrum), opinions tend to be more concentrated near one end, facilitating faster local consensus and fewer clusters. Conversely, when the initial mean is around the middle range, opinions are more evenly spread across the spectrum, increasing the likelihood of multiple distinct subgroups forming and thus more clusters emerge.

A real-world example of this dynamic can be seen in political landscapes. In a society where public opinion is largely polarized towards one extreme (e.g., strong support or opposition to a policy), consensus within groups may form quickly with limited fragmentation. However, when public opinion is more balanced and moderate across the spectrum, social groups often fragment into multiple factions with diverse viewpoints, reflecting a richer pluralism.

**(2) Impact of initial opinion variance on the evolution**

**Fig 6:**
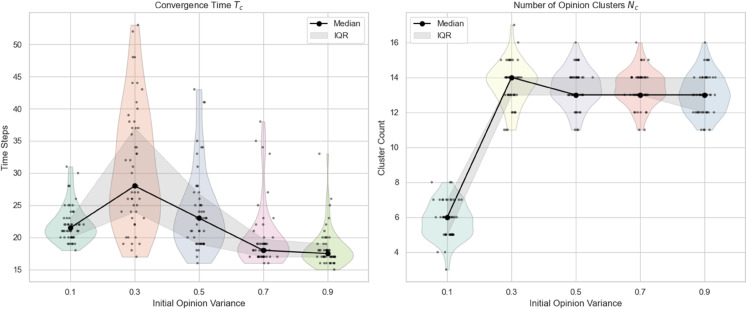
Comparison of convergence speed and cluster count under different initial opinion variance: The black dots represent the median of 100 repeated trials, and the shaded area represents the interquartile range (IQR).

In this part, we investigate the impact of the initial opinion variance on opinion evolution. Keeping all other parameters fixed, the initial opinion variance is varied across values of 0.1, 0.3, 0.5, 0.7, and 0.9. For each setting, 100 independent simulation runs are conducted to ensure the robustness of the results. This analysis aims to reveal how the diversity of initial opinions influences the convergence dynamics and the formation of opinion clusters. The results are shown in Fig 6.

Based on Fig 6, it can be observed that the convergence time exhibits a non-monotonic pattern with respect to the initial opinion variance. Specifically, as the variance increases, the convergence time initially rises, indicating slower opinion alignment due to increased opinion dispersion. However, beyond a certain threshold, the convergence time tends to stabilize within a relatively narrow range, showing only mild fluctuations.Interestingly, the variability in convergence time is more pronounced under moderate levels of opinion variance. This suggests that in such conditions, the system’s dynamics are more sensitive to stochastic factors, possibly due to the presence of multiple competing clusters that form and dissolve during the opinion evolution process. In contrast, both low and high variance scenarios show more stable and consistent convergence behaviors across simulation runs.

In comparison, the number of opinion clusters demonstrates a more abrupt, threshold-like transition. When the initial variance is low, most simulations converge to a small number of clusters, often close to consensus. However, once the variance exceeds a critical point, the number of clusters increases sharply and then remains relatively stable despite further increases in variance. This jump indicates a phase transition in the system’s ability to maintain connected opinion groups, beyond which fragmentation becomes dominant and persistent.

This behavior can be explained as follows: when initial opinions are very close to each other (low variance), agents tend to quickly reach consensus, resulting in fewer opinion clusters and longer convergence due to subtle opinion differences needing time to harmonize. As variance increases moderately, the opinion landscape becomes more diverse, creating distinct subgroups that rapidly form stable clusters, which reduces overall convergence time but increases cluster count sharply. However, when variance grows too large, some extreme opinions become isolated or less influential, causing fewer but more stable clusters, which slightly reduces the number of clusters.

**(3) Joint effects of initial opinion mean and variance**

**Fig 7:**
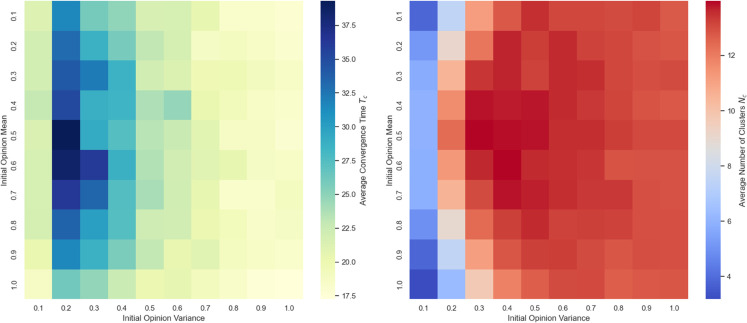
Heatmap of opinion convergence time and cluster number under varying initial opinion mean and variance: The vertical axis represents the mean of opinions, and the horizontal axis represents the variance of opinions. Darker colors indicate higher values.

In this section, we investigate the joint effects of the initial opinion mean and variance on opinion evolution. By systematically varying both the mean and variance from 0.1 to 0.9 with a step size of 0.1, while keeping other parameters fixed, we conducted 100 independent simulation runs for each parameter combination. The average convergence time and the average number of opinion clusters across these runs are visualized through heatmaps, which are summarized in the heatmaps shown in Fig 7, illustrating the combined influence of initial opinion mean and variance on convergence time and cluster formation.

As shown in Fig 7, the convergence time and the number of opinion clusters exhibit distinct patterns under varying combinations of initial opinion mean and variance. Regarding convergence speed, the initial opinion mean has little overall impact. Instead, the convergence time is primarily influenced by the variance. Across all levels of the mean, the longest convergence times occur when the variance is around 0.2. As the variance increases beyond this point, the convergence time decreases—a pattern consistent with the results observed in Fig 7.

In terms of the number of clusters, the influence of the opinion mean becomes more noticeable under low-variance conditions. Specifically, when the variance is small, moderate opinion means (e.g., around 0.5) tend to lead to a higher number of clusters, while both low and high means are associated with fewer clusters. Conversely, under high-variance conditions, changes in the opinion mean have only a marginal effect on the number of clusters.

These patterns can be interpreted through the lens of initial diversity and centrality in opinion distribution. When the variance is low, individuals’ initial opinions are closely clustered around the mean, making the position of that mean more influential in determining how opinions diverge or converge. For example, if the mean is near an extreme, most individuals already share a relatively similar opinion, facilitating faster consensus and resulting in fewer clusters. However, when the mean is moderate, even a low variance can still result in more ideological spread, which in turn increases the chances of fragmentation into multiple clusters. In contrast, under high-variance conditions, opinions are widely dispersed regardless of the mean. This inherent diversity leads to multiple stable clusters forming early in the dynamics, making the specific value of the mean less relevant.

**(4) Impact of group size on opinion evolution**

**Fig 8:**
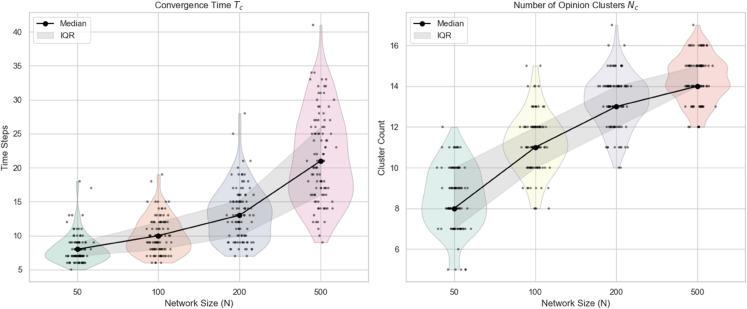
Comparison of convergence speed and cluster count under different group size: The black dots represent the median of 100 repeated trials, and the shaded area represents the interquartile range (IQR).

In this section, we investigate how the size of the population influences the evolution of opinions. To this end, we vary the number of individuals in the system by setting N=50,100,200,500, while keeping all other parameters fixed. For each group size, the experiment is repeated 100 times to obtain statistically robust results regarding convergence speed and the number of resulting opinion clusters. The results are shown in Fig 8.

From Fig 8, it can be find that as group size increases, both the convergence time and the number of opinion clusters exhibit a clear upward trend. This phenomenon can be attributed to the growing complexity of interactions within larger populations. In a larger group, individuals are exposed to a more diverse set of opinions, which makes it more difficult for the system to reach consensus quickly. Additionally, the increased diversity creates more opportunities for stable subgroups to form, leading to a higher number of opinion clusters.

**(5) Impact of weight allocation on opinion evolution**

**Fig 9:**
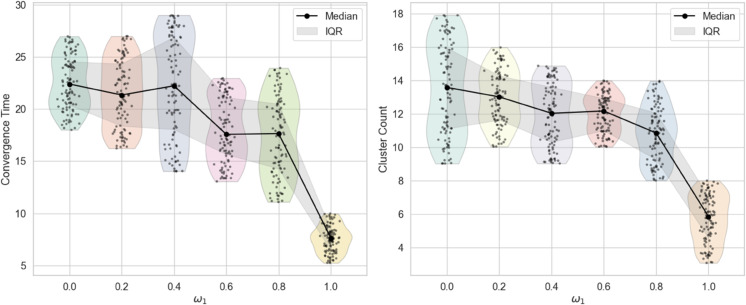
Comparison of convergence speed and cluster count under different weight allocation: The black dots represent the median of 100 repeated trials, and the shaded area represents the interquartile range (IQR).

In this section, we examine the effect of the weight allocation between opinion similarity (*w*_1_) and attribute similarity (*w*_2_) on the evolution of opinions. Specifically, *w*_1_ is set to 0, 0.2, 0.4, 0.6, 0.8, and 1, with $w_{2}=1-w_{1}$. Notably, when *w*_1_ = 1, the model reduces to the traditional HK model implemented on a BA network. The resulting opinion dynamics and clustering patterns for these parameter settings are presented in Fig 9, illustrating how different emphases on opinion similarity influence the collective behavior of the system.

As illustrated in Fig 9, increasing the weight *w*_1_ assigned to opinion similarity results in both the cluster count and the convergence time remaining largely stable, showing only a marginal downward trend. Notably, when *w*_1_ = 1, both quantities experience a pronounced decline, indicating a faster convergence and fewer final opinion clusters.

This phenomenon can be explained by the role of attribute similarity in the interaction dynamics. When *w*_1_<1, both opinion similarity and attribute similarity jointly determine the interaction strength between individuals. Agents update their opinions by considering not only the opinions of others but also their inherent attributes, which maintains diverse interactions and stabilizes the overall convergence behavior. In contrast, when *w*_1_ = 1, attribute similarity is completely ignored, and interactions depend solely on opinion proximity. In this extreme case, the model effectively reduces to the traditional HK model on a BA network, resulting in accelerated consensus formation and a reduced number of clusters. This highlights the crucial role of attribute similarity in sustaining opinion diversity and prolonging convergence time.

### 4.4 Model validation through real-world data

To validate the effectiveness of the proposed model, we conduct an empirical analysis using a publicly available Douban movie rating dataset from Kaggle. Since different network sizes can naturally lead to different numbers of opinion clusters, a direct comparison of cluster counts lacks a consistent reference framework. Therefore, this section only analyzes the convergence time. For experimental analysis. The dataset is represented as


$$D=\left(x_{i},C_{x_{i}},T_{x_{i}},R_{x_{i}}\right),$$

where *x*_*i*_ denotes the *i*-th movie, $C_{x_{i}}$ represents the set of reviews for movie *x*_*i*_, $T_{x_{i}}$ represents the set of review timestamps for movie *x*_*i*_, and $R_{x_{i}}$ denotes the star ratings for each review. The data processing procedure is as follows:

1. For each movie, we calculate its average daily rating count, defined as the total number of ratings divided by the number of days on which ratings were received. This value is used as the input parameter *N* in the simulation model.
2. For movie *x*_*i*_ at time *t*, we compute the proportion of ratings with star levels 1–5:
  $$p_{k}\left(x_{i},t\right)=\frac{\left|\{r_{j}\in R_{x_{i}}:r_{j}=k\}\right|}{\left|C_{x_{i}}\right|},\;k=1,2,3,4,5.$$
3. We plot the time series of the proportions for each star level and calculate the convergence time, defined as
  $$T_{c}=\min\left\{t\;|\;\forall k,\;p_{k}\left(x_{i},t+K\right)-p_{k}\left(x_{i},t\right)\leq \vartheta \right\}.$$
  In accordance with the settings described earlier, the threshold is set to ϑ=0.05 and the continuous stability window is set to *K* = 5.

**Fig 10:**
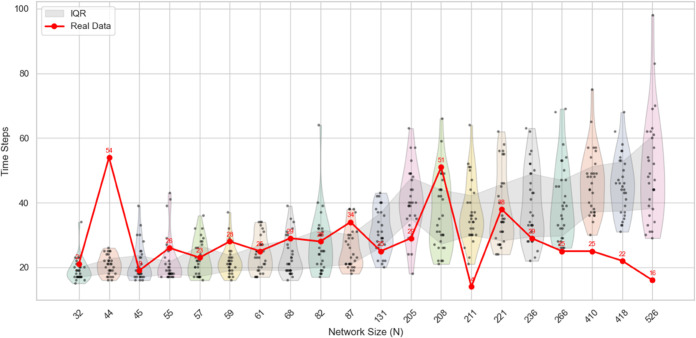
Comparison between simulated results of the proposed model and empirical observations: The red dots represent the real data points, the black dots represent the results of the proposed model from the simulated experiments, and the shaded area indicates the interquartile range (IQR).

**Fig 11:**
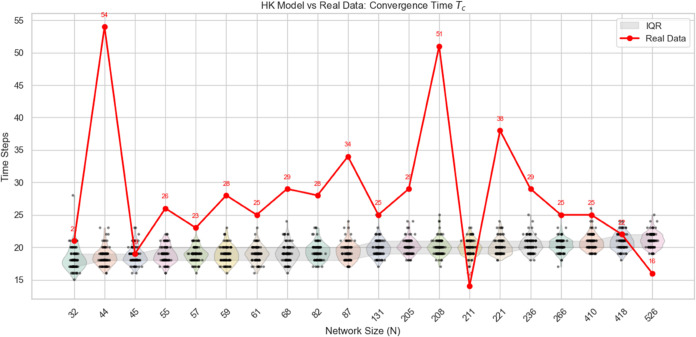
Comparison between simulated results of the proposed model and empirical observations: The red dots represent the real data points, the black dots represent the results of the HK model from the simulated experiments, and the shaded area indicates the interquartile range (IQR).

A comparison between the real and simulated convergence times is presented in Figs 10 and 11 .

As illustrated in Fig 10, the overall trend of the simulated convergence times aligns well with the empirical data, particularly in scenarios involving smaller population sizes. This consistency indicates that the model effectively captures the underlying dynamics governing opinion convergence when the number of participants is limited. However, as the group size increases, the simulation results exhibit greater variability and begin to deviate from the real-world observations. This discrepancy suggests potential limitations of the model in accurately replicating convergence behavior in large-scale social interactions. One possible explanation for this deviation lies in the inherent complexity of high-participation discussion environments, which may involve a broader spectrum of opinions, heightened emotional dynamics, and more heterogeneous interaction patterns.

Additionally, the empirical data—sourced from movie-related discussions—may be influenced by contextual factors such as varying engagement intensity, sentiment volatility, and uneven temporal distribution of comments. These factors introduce noise and structural irregularities that the current modeling framework may not fully account for, thereby affecting its predictive accuracy in such settings. However, compared to traditional models, the current model demonstrates higher predictive accuracy. Traditional models struggle to capture the complex social interaction effects characteristic of the information age. In contrast, our model, through more refined simulation and optimization, can more accurately reflect the impact of varying participant numbers on convergence time, especially under lower participation scenarios where its predictions align more closely with empirical data. Therefore, despite certain limitations, the proposed model significantly outperforms conventional approaches, offering greater predictive precision and better adaptability.

**Fig 12:**
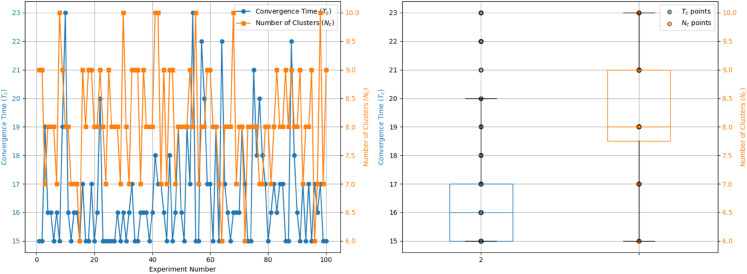
Comparison between simulated results of HK model with ε=0.05 and empirical observations: In the left figure, the yellow and blue dots respectively represent the convergence time and the number of clusters obtained from 100 repeated experiments, while the right figure shows the corresponding distributions.

To further verify that the observed differences are indeed due to the proposed model rather than arbitrary parameter settings, we conducted a sensitivity analysis of the traditional H-K model. In the current setting, the bounded confidence threshold *ε* is fixed at 0.1. Increasing this threshold produces two clear effects: (1) in terms of convergence speed, more individuals adjust their opinions in each time step, leading to faster convergence; and (2) in terms of clustering, the number of clusters decreases and cannot increase. Therefore, only decreasing *ε* could potentially make the H-K results closer to the empirical observations. We have conducted additional simulations with ε=0.05, as shown in Fig 12.

The results indicate that, although the convergence time becomes closer to the empirical data (*T*_*c*_>20), some differences still remain. Only further adjustments could make the convergence time fully align with the real data. Moreover, we note that existing literature rarely employs such a low threshold value. Taken together, these findings confirm that adjusting *ε* alone is insufficient to replicate the observed clustering patterns or to achieve better correspondence with the empirical results. This suggests that the differences we observe are not simply a consequence of parameter manipulation in the original HK model, but rather stem from the extended features introduced in our model.

## 5 Conclusion

In response to the growing complexity of opinion dissemination in the information era, this paper proposed an enhanced opinion dynamics model that integrates individual heterogeneity, domain-specific attributes, and multi-layer influence propagation mechanisms. Building upon the classical HK framework, the model redefines the influence neighborhood by incorporating the structural constraints of social networks, thereby capturing selective interaction behaviors observed in real-world settings. Additionally, influence weights are modeled based on homophily theory to reflect the tendency of individuals to be influenced by others with similar views. The model further introduces a *k*-layer influence propagation mechanism to simulate the non-local transmission of information within social networks. Simulation results demonstrate that the initial mean and variance of opinions significantly impact convergence speed and clustering structure. Moreover, as group size increases, convergence time tends to grow while opinion fragmentation becomes more pronounced. In environments characterized by high heterogeneity or deep influence transmission paths, oscillatory behaviors and coexistence of local stable states frequently emerge, indicating that opinion evolution is jointly driven by individual-level traits and network-level influence dynamics.

These findings offer both theoretical insights and practical value for applications such as public opinion modeling, crisis response, and online community governance. Understanding the mechanisms and trajectories of opinion evolution can facilitate real-time monitoring and predictive assessment of collective sentiment. However, several limitations remain. First, the current model does not incorporate psychological factors such as emotional fluctuations or information credibility, which may constrain its applicability to certain real-world scenarios. Second, the validation is based primarily on synthetic data and a limited set of empirical observations, requiring further testing across larger and more diverse datasets. Future work will focus on three key directions: (1) incorporating dynamic emotional and cognitive processes to enhance behavioral realism; (2) integrating heterogeneous, multi-source social data to improve model calibration and predictive capacity; and (3) developing policy-oriented simulation tools to explore how targeted interventions—such as modifying information pathways or restructuring social ties—can be employed to guide opinion trajectories and support evidence-based decision-making.
